# Child Skeletal Fluorosis from Indoor Burning of Coal in Southwestern China

**DOI:** 10.1155/2009/969764

**Published:** 2009-10-19

**Authors:** Xianghui Qin, Shouying Wang, Maojuan Yu, Lei Zhang, Xinhua Li, Zhen Zuo, Xiuhui Zhang, Lihua Wang

**Affiliations:** ^1^Department of Preventive Medicine, Guiyang Medical College, School of Public Health, Guiyang, Guizhou 550004, China; ^2^Office of Health Data and Research, Mississippi State Department of Health, P. O. Box 1700, Jackson, MS 39215-1700, USA

## Abstract

*Objectives*. We assess the prevalence and pathogenic stage of skeletal fluorosis among children and adolescents residing in a severe coal-burning endemic fluorosis area of southwest China. *Methods*. We used a cross-sectional design. A total of 1,616 students aged between 7 and 16 years in Zhijin County, Guizhou, China in late 2004 were selected via a cluster sampling of all 9-year compulsory education schools to complete the study questionnaire. Any student lived in a household that burned coal, used an open-burning stove, or baked foodstuffs over a coal stove was deemed high-risk for skeletal fluorosis. About 23% (370) of students (188 boys, 182 girls) were identified as high-risk and further examined by X-ray. *Results*. One-third of the 370 high-risk participants were diagnosed with skeletal fluorosis. Overall prevalence of child skeletal fluorosis due to indoor burning of coal was 7.5%. Children aged 12–16 years were significantly more likely to be diagnosed with skeletal fluorosis than children aged 7–11 years (OR = 1.84, 95% CI: 1.17–2.90; *P* = .0082). Four types of skeletal fluorosis were identified: constrictive (60.7%), raritas (15.6%), mixed (16.4%), and soft (7.4%). Most diagnosed cases (91%) were mild or moderate in severity. In addition, about 97% of 370 high-risk children were identified with dental fluorosis. Dental fluorosis was highly correlated with skeletal fluorosis in this study. *Conclusions*. Skeletal fluorosis among children may contribute to poor health and reduced productivity when they reach adulthood. Further efforts to reduce fluoride exposure among children in southwestern of China where coal is burned indoors are desperately needed.

## 1. Introduction

Fluoride has both positive and negative effects on human health. In fact, fluoride has been shown to prevent dental caries mainly through fluoride's topical action. Conversely, chronic exposure to excessive amounts of fluoride may cause an abnormal condition called skeletal fluorosis. Overexposure to environmental fluoride has caused endemic fluorosis to become a major public health problem in China. Endemic fluorosis is prevalent in 29 provinces, municipalities, or autonomous regions in China and is associated with three types of fluoride exposure: contaminated drinking water, pollution from coal burning, and tea drinking. More than 10 million people in Guizhou Province suffer from various forms of fluorosis [1, 2]. In the southwestern province of Guizhou, endemic fluorosis has been attributed to the use of fluoride-rich coal as an indoor fuel source [1–6]. Coal is the main fuel used by the local inhabitants of Zhijin County. Zhijin is a mountainous area where the moist climate requires residents to burn coal stoves year round in order to dry corn, a dietary staple. Similar to other peasants in this region of China, the local inhabitants use open-burning style stoves, without adequate ventilation, to bake food or dry corn hanging over the stove. Fluoride is released from the coal and accumulates in the drying corn, significantly increasing the amount of fluoride a family member is exposed to upon consumption [7]. In 1989, ZhengandHuang hypothesized that fluoride absorbed by corn dried over coal-burning stoves was the probable cause of endemic fluorosis in this region [1]. Families also burn coal to heat their homes. Gaseous forms of fluoride, such as hydrogen fluoride (HF) or silicon tetrafluoride (SiF_4_), and fluoride dust also pollute indoor air in homes and may be inhaled directly or further concentrate in drying foodstuffs [8]. Average family coal consumption is estimated to be 2–3 tons annually. Some families even burn more than 500 kg in 15 days [9].

Skeletal fluorosis (SF) is a chronic metabolic bone disease that occurs when abnormally large amounts of fluoride accumulate in bone tissue. Because bone growth and remodeling occur over a human's lifespan, SF can progressively worsen with overexposure to high fluoride levels [10]. A variety of conditions are associated with SF including osteosclerosis, osteomalacia, osteoporosis, and secondary hyperparathyroidism; severity is also varied, ranging from mild joint pain to crippling disabilities and severe bone deformations [10, 11]. Typical signs and symptoms include aches and pains, limited joint movement, knock-knees, bowing of legs, and spinal curvature. Clinical symptoms may not be present until SF cases become advanced. Radiological changes are discernable at earlier stages of disease revealing irregular thickening and high density of bones upon X-ray examination [10].

Epidemiological studies have historically focused on SF among adults residing in fluorosis endemic areas [3, 11–14]. Adults living in these areas should theoretically have longer cumulative exposure to excessive levels of fluoride from their environments and thus higher prevalence than children. Despite limited lifetime exposure to fluoride among juvenile populations, children living in endemic fluorosis areas also suffer from SF [15, 16]. The effects of various levels of fluoride intake, specifically persistent exposure to high levels of fluoride, on rapidly developing bones in young children are not well understood. Fluoride can alter accretion and resorption of bone tissue or interrupt the homeostasis of mineral metabolism in bone [16]. Increased metabolic turnover of bone, impaired synthesis of bone, or fluoride accumulation in the crystal lattice of skeletal tissue may all be important mechanisms for how fluoride affects bone tissue in rapidly growing children. Furthermore, only a few endemic fluorosis studies have focused on the prevalence of SF among children or adolescents [16, 17]. Previous reports of juvenile SF in southwestern China suggest that children diagnosed with SF tend to be young, exhibit bone deformations, and have X-ray signs of mixed type bone transformation [17]. A more in-depth understanding of the prevalence and severity of SF among juvenile populations is required in order to plan control efforts and reduce fluorosis-related disease and disability in highly endemic areas.

Dental fluorosis (DF) is a permanent hypomineralization of tooth enamel caused by an overexposure to fluoride during tooth development. DF affects teeth during permanent tooth formation, which usually occurs in childhood between the ages of 6 months to 5 years. After permanent teeth erupt into the oral cavity, signs of DF are considered permanent without cosmetic dental repair. Typical signs of DF include yellowing of teeth, white spots, mottling of enamel, and brown staining in severe cases. Enamel damage resulting from DF can be viewed on the permanent teeth of children as well as adults. Accordingly, epidemiology of endemic fluorosis among children is commonly reported in terms of prevalence and severity of DF of children living in endemic fluorosis regions [2, 4, 10, 11]. Although other investigations have reported on the prevalence of DF in regions of China, none have focused on our area of interest, Zhijin County. Since fluorine exposure is highly correlated to the immediate environment in which a person lives, specific estimates for Zhijin County are necessary in order to understand the extent and severity of endemic fluorosis affecting this area.

The present study is among the first to report SF prevalence among children/adolescents living in a coal-burning endemic fluorosis area. It was designed to (1) assess the prevalence of SF and DF among children from indoor burning of coal, and (2) describe the pathogenic state of SF among children and adolescents residing in a severe endemic fluorosis area.

## 2. Methods

### 2.1. Design

A cross-sectional survey was conducted in a rural, heavily polluted region of Zhijin County, Guizhou, China, between October and December 2004.

### 2.2. Participants

Participants included children who were studying at all the 9-year compulsory education schools in the rural area: Guohua, Hehua, Lianhe, Niotong, and Yanzai primary schools. Students attending these five schools have very similar living conditions and differ minimally in terms of lifestyle, parental education level, socioeconomic status, and medical care. The study proposal was approved by the Guiyang Medical College Internal Review Board.

### 2.3. Sampling

To save time and reduce the cost, a cluster sample design was used. It produced a representative sample of students in grades 1–9 who enrolled in the five choosing primary schools. A total of 1616 students aged 7–16 years participated the study. Data collection took place in the schools during the period of Education Examination by Guizhou Bureau of Education, a time when schools typically have high attendance rates. The study investigation team and primary school teachers supervised children during data collection. Screening questions were explained to the children in the younger age range at the time of the survey. Selected students completed a questionnaire that included questions about their lifestyle and exposure to burning coal. For this study, living in a household that (1) burned coal, (2) used an open-burning style stove, or (3) baked/dried foodstuffs over a coal stove was considered an SF risk factor. Based upon responses, 370 students were classified as high-risk for SF and further examined for SF.

### 2.4. Measures

#### 2.4.1. Diagnosis of Skeletal Fluorosis

Children were examined and X-rays were taken at their own school. Directed by the technologist of the Radiology Department of Guiyang Medical College, X-rays of each child's pelvis region and lower limbs were taken by the technician of the Radiology Department of Zhijin Traditional Chinese Medical Hospital. An endemic SF specialist examined all X-ray films and made official diagnoses. X-ray checkpoints were the pelvis and right lower limbs (ankles were included, if necessary). Diagnoses were made according to the Chinese standards for Radiological Diagnosis of Skeletal Fluorosis, published by the Chinese Department of Health on November 26, 1999 (professional standard number: WS 192-1999). The Chinese standard is line with the international standard first propose by Roholm in 1937, then modified by Singh and Jolly in 1970. Diagnosed cases were divided into three grades mild, moderate, and severe and four types: constrictive, raritas, mixed, and soft. The grade of SF was further defined using radiographic characteristics from hyperostosis, osteopenia, and turn over of bone. Detailed descriptions on 33 criteria for grading were illustrated in Appendix A of the WS 192-1999.

#### 2.4.2. Definition of Skeletal Fluorosis Type

Constrictive type SF is characterized by a thickening of bone trabeculae in the pelvis. Bone spots, dense patches, and os in os phenomenon in the pelvic region are also observed with this type of SF. Children with constrictive type may also have multiple growth retardation lines in the long bones of their legs or lateral line-shaped shadows in the compact bone trabeculae in the leg metaphysis [17]. Raritas type SF shows less thickening of the bone trabeculae in the pelvis; bone trabeculae are thinning or rare in the metaphysis of the legs. Cortical bone thickening and lower densities in the tibiofibular region also characterize raritas type. With raritas type, strip microtubule lucency lines are increasingly visible. Mixed type SF is diagnosed when constrictive and raritas types exist at the same time. Soft type SF is characterized by lower density of the bony structure, thickening of bone trabeculae, raritas, and veil construction. With soft type, tibia bones may be abnormally curved. Pelvic deformity and os in os phenomenon in the abdominal vertebra are also associated with soft type SF.

#### 2.4.3. Definition of Dental Fluorosis

Dean's Scale was used to assign and record a dental fluorosis index score for each child [15]. DF classifications for this investigation included normal, questionable, very mild, mild, moderate or severe. Normal teeth appeared smooth and glossy with a white translucent surface. A few white specks on tooth enamel were classified as questionable. In contrast, DF appeared as opaque white areas on the tooth surface. Moderate cases typically affected the entire tooth surface and a brown stain may have been present. In severe cases, the entire tooth surface was affected, enamel pitting was present, and brown staining was evident. Prevalence of DF was based upon index scores of very mild to severe. Index scores were assigned ordinal values as follows: normal or questionable = 0, very mild = 1, mild = 2, moderate = 3, and severe = 4.

### 2.5. Statistical Analysis

SPSS 11.5 (SPSS Inc., Chicago, Ill, USA) was used for all data management and analysis. Chi-square tests were used to assess the statistical significance of observed differences in disease prevalence or SF type according to gender or age. Odds ratios (ORs) and 95% confidence intervals (CIs) were calculated for all2x2bivariate associations. Chi-square tests were also used to assess the observed differences and a linear trend in disease grade by gender. All reported *P*-values are two-sided and *P* < .05 was considered statistically significant.

## 3. Results

Table 1 depicts SF detection rates by gender. Among the 370 high-risk children, 122 were diagnosed with SF. Data collected from the sampled children (1616) were used to estimate the current prevalence of SF in the study population. In this severe endemic fluorosis area, the prevalence of SF among children ages 7 to 16 years from indoor burning of coal was 7.5%. In addition, boys were 48% more likely to be diagnosed compared to girls, although the higher proportion was of marginal significance (OR = 1.48, 95% CI: 0.96–2.30; *P* = .0764). Table 2 illustrates SF detection rates by gender and age. Older children (12–16 years) were significantly more likely to be diagnosed with SF compared to younger children (OR = 1.84, 95% CI: 1.17–2.90; *P* = .0082).

**Figure 1:**
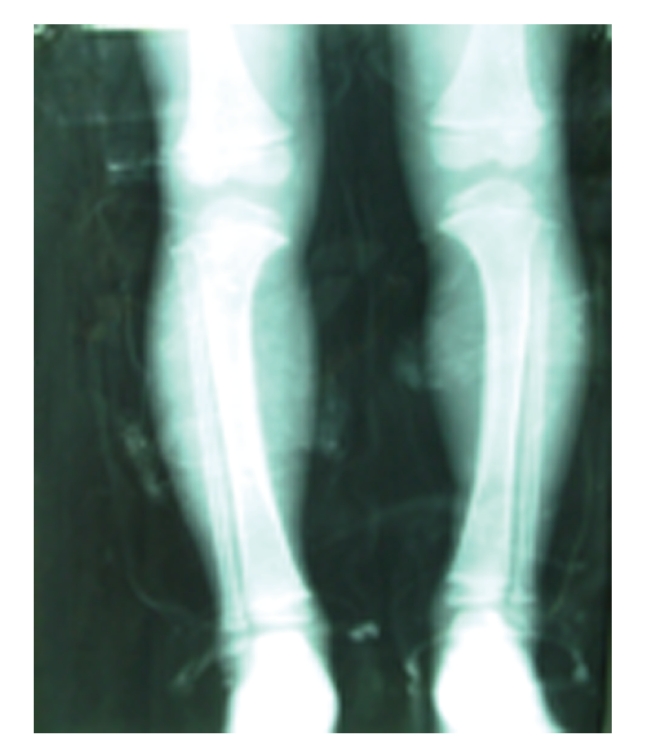
X-ray for a 7-year old boy.

**Figure 2:**
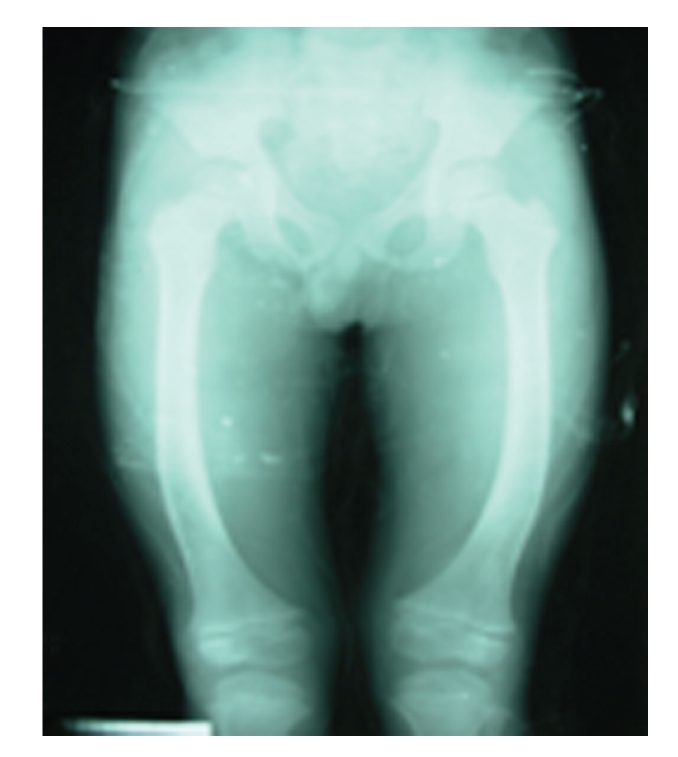
X-ray for another 7-year old boy.

Table 3 describes the distribution of SF type and grade among the affected children. Constrictive type was the most common form of SF in Zhijin County, affecting 74 (60.7%) children. Raritas and mixed-type SF accounted for 39 cases (32.0%); soft type was the least common form of the disease. SF type did not appear to differ according to gender. Of the 122 SF cases, 111 (91.0%) were classified as mild or moderate in severity. Severe SF was diagnosed in 8 boys and 3 girls. Two of these children, both 7 years old, had severe skeletal deformities (X-shaped or O- shaped legs, Figures 1 and 2). The degree of severity did not appear increase with age (data not shown). On average, SF severity was worse (grade was higher) among boys compared to girls; however, severity differences according to gender were not statistically significant (*P* = .396). In addition, no significant linear trend across grade of SF by gender was observed (*P* = .183).

All 370 children, regardless of SF disease status, were screened for DF. A total of 358 children (96.8%) were diagnosed with some form of DF (Table 4). Boys were slightly more likely to be diagnosed with DF than girls (data not shown). Only 10 (2.7%) children had normal teeth with no signs of fluorosis. Two children were diagnosed as questionable and not included in the DF prevalence estimates. Nearly all (99.2%) of children with SF were also diagnosed with DF, while 95.6% of children without SF showed clinical signs of DF. In our sample, the majority of Zhijin County children had either moderate or severe DF index scores.

## 4. Discussion

This study provides important evidence on the percentage of SF among children and adolescents in southwest China and creates a baseline to assess future endemic fluorosis control efforts in this region. As expected, percentage of SF was higher among older children compared to children under 12 years old, but no gender differences were observed. Constrictive-type SF was the most common type of SF although raritas, mixed, and soft type were also diagnosed. Nearly half of children with SF were classified as moderate and 9% suffered from severe SF.

The strengths of this study include methodological steps taken to ensure that the estimates were valid and representative of children in this area and all SF diagnoses were made consistently. All children attending every one of the compulsory 9-year schools in the region were included in the sampling frame. Data collection occurred during the school year at local schools and coincided with mandatory exams. School attendance rates are at a peak during this time of the school year, helping to ensure high participation rates. To ensure consistent and valid disease diagnosis, a single experienced technician took X-ray photos of all 370 high-risk children and one experienced SF expert analyzed all X-ray photos and made official SF diagnoses.

The study has several weaknesses or limitations worthy of comment. A survey can only assess disease distribution in a population at one moment in time. SF occurs as a result of cumulative exposure to excess fluoride levels and a minimum threshold of exposure has yet to be determined. Thus, students that showed no X-ray signs of SF during our study could conceivably be diagnosed as mild cases six months later. Furthermore, X-ray diagnosis is expensive and individual diagnosis of the entire study population was not feasible. It is possible that students at low risk for SF, at the time of the study, indeed would have been diagnosed with SF if examined further by X-ray. Thus, our estimate for the entire study population is likely to be lower than the true prevalence among primary school children in Zhijin County. SF diagnoses, type identifications, and grade classifications were assigned by one SF expert. While this design feature ensures that all children were diagnosed under the same study criteria, while repeatability can be assessed, any investigator bias will likely be included in the reported results. Also worthy of mention is that disease grade or severity was assessed on a qualitative ordinal scale of mild, moderate, or severe. These categories are open to diagnostic interpretation when SF is subclinical or asymptomatic and participant interpretation when disease is clinical. The degree to which grade misclassification occurred is unknown but expected to be minimal.

This study did not assess the source of fluoride exposure among residents of Zhijin County. Recent studies in literature have assessed the source of fluoride exposure in the southwestern provinces of China and concluded that the majority of fluoride exposure occurs as a result of indoor coal burning [1, 4, 6, 18, 19]. Some regions in southwestern China burn pure high fluoride coal, while in other regions the fluoride levels in coal are compounded by high fluoride levels in clay used as a binder for coal briquettes [1, 6]. In 2004, Dai et al. showed that most coals from western Guizhou Province were actually low fluoride coal, with a fluoride concentration of less than 200 ppm, and reported that average fluoride content of coal in Zhijin county was 89 ppm [19]. However, residents commonly used yellow clay that contained high fluoride content of 324 ppm as a coal-burning additive in furnaces or as a binder in briquette making [19]. The daily primary fuel among residents in the present study is a mixture of coal and clay. Other residents may use solid coal, but many still moisten and grind coal into a paste and subsequently mix it with clay to form briquettes for combustion [18].

Ando et al. considered how fluoride levels in coals from Sichuan Province could result in overexposure to fluoride and later cause fluorosis among local residents [4]. Upon burning, gaseous fluoride and particulate dust-containing fluoride are released from coal briquettes. Ando et al. estimated that only 2% of fluoride exposure was due to inhalation of indoor air in coal- burning households, while 97% of fluoride exposure was due to ingestion of food dried and stored inside the household [4]. Residents of Zhij in in Guizhou Province cook foodstuffs in the same fashion as residents of Sichuan Province. Corn and potatoes are the primary food consumed by residents of Zhij in. Corn is hung over open style coal-burning stoves to dry. Wet corn absorbs fluoride volatilized by burning coal [4, 18]. Ando et al. measured fluoride content in corn dried over coal-burning stoves. Daily mean fluoride intake (per person) from corn was 37.6 mg and 3.5 mg from potatoes [4]. More importantly, future research should investigate the level of fluoride intake necessary for the development of skeletal fluorosis in pediatric populations.

The two other sources of overexposure to fluoride in China are tea drinking and drinking water. Brick tea drinking is not a customary practice among children in this region of China, so fluoride exposure from this source is unlikely. Results from previous investigations have ruled out contaminated drinking water as the main source of endemic fluorosis in Zhijin County [4]. One recent study assessed fluoride levels in the drinking water of the two most severe endemic fluorosis villages in Zhijin County, Hehua and Hualuo. Average fluoride levels in the villages' drinking water is well below the World Health Organization (WHO) guideline value for drinking water [19, 20]. While drinking water levels may not be the main source of fluoride exposure, they should be considered in efforts to reduce fluoride exposure below a certain threshold. Endemic fluorosis develops in response to total fluoride intake. Thus, exposure to all sources of fluoride should be assessed if control measures are to be successful.

Two other studies have reported average fluoride intake per day and skeletal radiographic appearances among children and adolescents with SF in Guizhou province; however, both studies conducted in the late 1980s were small (less than 70 participants) and none of them investigated SF prevalence [17, 21]. Only one large study in China has reported SF prevalence among children. In 1998, Ando et al. investigated SF prevalence among residents of a neighboring coal-burning endemic fluorosis area, Pengsui County in Sichuan Province [4]. However, due to the lapse of time between studies (almost 10 years) and differences in sampling and SF diagnosis criteria, all comparisons between children in the two provinces should be speculative. The present study assessed SF in a high-risk juvenile population, while the Sichuan Province study assessed a sample of residents from the general population, which included children and adolescents. More important than direct comparison of the two provinces is the fact that in two serious endemic coal-burning fluorosis areas, at two different time points in the recent past, children and adolescents were experiencing SF signs and symptoms.

Severe bone deformities, resulting from endemic fluorosis, have also been identified in children who live in India. In a recent study, skeletal signs of SF, including Genu valgum, Genu varum, and anterior bowing of legs, were identified in children ages 1–18 years, who resided in endemic fluorosis villages of the Bihar State, India [16]. Study investigators concluded that high rates of SF among children/adolescents in this region are due to overexposure to high fluoride levels coupled with serious vitamin and mineral deficiencies, especially calcium and vitamin D deficiencies [16]. One limitation of the present study is that we did not measure vitamin and mineral intake among our children. It is possible that the severity of skeletal fluorosis cases among children in Zhijin may vary due to other factors that could include the child's diet and intake of vitamins and minerals necessary for proper bone growth and development. Further investigation is necessary to assess all the potential factors that contribute to SF incidence and severity among children and adolescents in China. While excess fluoride exposure is necessary to cause SF, we expect that SF severity in pediatric populations is related to other contributing factors such as dietary intake, exercise, concurrent diseases, and general overall health.

A staggering 97% of children from our sample had some clinical signs of DF. Furthermore, nearly 84% of these children had signs of moderate-to-severe fluorosis on at least one tooth. DF prevalence among Zhijin children/adolescents in our sample is similar to the prevalence among children, aged 10–15 years, in Pengsui County, Sichuan Province, where 99.5% of children present with signs of DF [4]. Extremely high prevalence of DF among children/adolescents suggests that exposure to high fluorine levels in these areas of southwestern China has been occurring in the recent past (as tooth development occurs early in a child's lifespan). Animal studies suggest that teeth may be good biomarkers for skeletal fluoride exposure in humans exposed to greater than optimal levels of fluoride [22]. Hence, children and adolescent populations with high DF incidence rates may represent at risk populations for SF in adulthood and further highlights the need for fluorosis control measures and de-fluoridation projects in southwestern regions of China.

The present study is one of very few to report percentage of SF among children living in a severely endemic fluorosis region of China. Often excluded, or under-sampled, in epidemiological reports of endemic fluorosis, prevalence of SF in children and adolescents closely represents the incidence of SF in a population and can be used to assess the successfulness of control measures at the population level.

China recognized fluorosis as a major endemic disease in 1977 and the Chinese Government has attached great importance to controlling fluorosis [23]. Pilot schemes designed to improve domestic stoves were launched in the 1980's and a campaign to adapt stoves in Guizhou Province was conducted in the 1990's [23]. During the campaign, open stoves were converted into closed stoves by the addition of a chimney. Despite improved household stoves, populations in Guizhou Province still suffer from high exposure to indoor air pollution, and adverse effects from overexposure to fluoride [22]. Recent studies have indicated that limiting inhalation of stove emissions by using a chimney-style stove would only result in a minor reduction in fluoride exposure [4]. New fuel sources or other methods of drying foodstuff would have a greater impact on fluoride exposure levels in this region of China. Other methods of fluoride exposure control need to be designed if fluorosis prevalence among residents of coal- burning endemic fluorosis areas is to be reduced.

## 5. Conclusions

Indoor burning of fluoride-rich coal is believed to cause endemic fluorosis cases among children in the Guizhou Province of China. The present study illuminates the extent of endemic SF among children and adolescents living in a heavily polluted neighborhood of Guizhou Province, Zhijin County. In our sample, 7.5% of the children were diagnosed radio graphically with SF.

Peasants in many regions of China depend upon coal as a fuel source because it is cheap and abundant. In many regions of China, both the coal and clay used to make coal briquettes (for burning) contain high amounts of naturally occurring fluoride. Past efforts to reduce fluoride exposure have included remodeling of stoves, sometimes to include a chimney to reduce indoor exhaust. Stove remodeling in this region of China has not curbed the prevalence of skeletal fluorosis among children. Further efforts to reduce chronic fluoride exposure among children and adolescents in this and other regions of China where coal is burned indoors are desperately needed.

## Figures and Tables

**Table 1:** Prevalence of skeletal fluorosis (SF) among children in a coal-burning endemic fluorosis area, Zhijin, Guizhou, China, 2004.

Gender	*n*	Number examined	SF cases detected	Prevalence (%)
Boys	830	188	70	8.4^§^
Girls	786	182	52	6.6

Total	1,616	370	122	7.5

^§^Boys were 1.48 times as likely as girls to be diagnosed with skeletal fluorosis (OR = 1.48, 95% CI: 0.96–2.30; *P* = .0764).

**Table 2:** Detection of skeletal fluorosis (SF) among 370 sampled children according to gender and age, Zhijin, Guizhou, China, 2004.

Age (yr)	Boys		Girls		Total	
	Number examined	SF *n* (%)	Number examined	SF *n* (%)	Number examined	SF *n* (%)
7–11	78	21 (26.9)	76	18 (23.7)	154	39 (25.3)
12–16	110	49 (44.5)	106	34 (32.1)	216	83 (33.4)^§^

Total	188	70 (37.2)	182	52 (28.6)	370	122 (33.0)

^§^Older children (12–16 years) were significantly more likely to be diagnosed with skeletal fluorosis than younger children (7–11 years) (OR = 1.84, 95% CI: 1.17-2.90; *P* = .0082).

**Table 3:** Distribution of type and grade of Skeletal fluorosis (SF) detected among children in a coal-burning endemic fluorosis area, Zhijin, Guizhou, China, 2004.

	Boys	Girls	Total
	*n* (%)	*n* (%)	*n* (%)
*Type of SF*			
Constrictive type	46 (37.7)	28 (23.0)	74 (60.7)
Raritas type	9 (7.4)	10 (8.2)	19 (15.6)
Mixed type	8 (6.6)	12 (9.8)	20 (16.4)
Soft type	7 (5.7)	2 (1.6)	9 (7.4)

Total	70 (57.4)	52 (42.6)^§^	122 (100.0)

*Grade of SF*			
Mild	28 (23.0)	26 (21.3)	54 (44.3)
Moderate	34 (27.9)	23 (18.8)	57 (46.7)
Severe	8 (6.6)	3 (2.5)	11 (9.0)

Total	70 (57.4)	52 (42.6)^†^	122 (100.0)

^§^Type of SF does not differ by gender (*χ* ^2^ = 5.472, df = 3; *P* = .140).

^†^Grade of SF does not differ by gender (*χ* ^2^ = 1.854, df = 2; *P* = .396).

**Table 4:** Prevalence of Dental Fluorosis (DF) and distribution of Dean's Fluorosis Index Scores among 370 sampled children in a coal-burning endemic fluorosis area, Zhijin, Guizhou, China, 2004.

Skeletal Fluorosis Status	Dean's Fluorosis Index Scores							Prevalence^§^ of DF
		Normal	Questionable	Very mild	Mild	Moderate	Severe	
	*n*	*n* (%)	*n* (%)	*n* (%)	*n* (%)	*n* (%)	*n* (%)	(%)
Yes	122	0	1 (0.3)	3 (0.8)	9 (2.4)	71 (19.2)	38 (10.3)	99.2
No	248	10 (2.7)	1 (0.3)	4 (1.1)	32 (8.7)	130 (35.1)	71 (19.2)	95.6
Total	370	10 (2.7)	2 (0.5)	7 (1.9)	41 (11.1)	201 (54.3)	109 (29.5)	96.8

^§^Data do not include questionable DF cases.
